# Lower socioeconomic status strengthens the risk of otitis media with effusion after COVID-19 infection: a case-crossover study in Northeast China

**DOI:** 10.1038/s41598-026-61832-9

**Published:** 2026-07-09

**Authors:** Ruiyang Ma, Ying Tian, Wei Shao, Jiaxing Guo, Shuhan Li, Fei Yu, Lifeng Zhang, Shuai Hao

**Affiliations:** 1 Department of Otolaryngology, First Affiliated Hospital of China Medical University, No. 155, Nanjing North Street, Heping District, Shenyang, 110001 China; 2 Liaoning Institute of Basic Medical Sciences, No. 2, Qiaosong Road, Su Jiatun District, Shenyang, 110101 China; 3 Department of Maternal, Child & Adolescent Health, School of Public Health, Shenyang Medical College, Shenyang, 110034 China

**Keywords:** Socioeconomic status, Otitis media with effusion, COVID-19, Socioeconomic scenarios, Infectious diseases

## Abstract

To investigate the associations of otitis media with effusion (OME) with different socioeconomic status (SES) before during and after the coronavirus disease 2019 (COVID-19) outbreak in Northeast China. Three cross-sectional data on daily emergency and outpatient were included pre, during and post COVID-19 epidemic. We recorded the number of OME and symptoms and confirmed SARS-Cov-2 infection in patients with OME. We used logistic regression to evaluate the odds ratio (OR) for OME with COVID-19 infection, and employed an interrupted time series analysis to quantify the effect of COVID-19 outbreak on OME, and a time-stratified case-crossover analysis to evaluate the association of OME with SES before, during and after the COVID-19 outbreak, especially in the two months following the COVID-19 outbreak. During COVID-19 outbreak, the OME significantly increased by 50% (RR: 1.563, 95% CI 1.256–1.942) compared to the same period in other years, low SES was associated with an increased risk of OME, but this low SES-related risk elevation significantly increased during the outbreak of COVID-19, especially in the first month after COVID-19 infection. Individuals under 50 years old were still vulnerable to SES after COVID-19 infection in Liaoning. Lower SES might aggravate OME and greater efforts are needed to improve SES in post-COVID-19.

**Supplementary Information:**

The online version contains supplementary material available at 10.1038/s41598-026-61832-9.

## Introduction

The coronavirus disease 2019 (COVID-19) initially occurred in Wuhan (China) in December 2019^1^. Until January 2024, COVID-19 has infected over 774 million people and caused over 7.0 million deaths globally^2^. The COVID-19 pandemic became public health emergency of international concern caused by severe acute respiratory syndrome coronavirus 2 (SARS-CoV-2) in March 2020. SARS-CoV-2 continued to mutate, with a new mutant strain Omicron becoming the main prevalent strain in China since October 2022^3^. Omicron mainly causes changes in clinical manifestations, including a decrease in pneumonia symptoms and an increase in upper respiratory tract infections, especially the eustachian tube infection^4^. Upper respiratory tract infections often lead to middle ear infections^5^. SARS-CoV-2 is found in the middle ear fluid (MEF) of patients with otitis media with effusion (OME)^6^. The OME patients were previously COVID-19-positive, OME symptoms may still appear even after the second or third COVID-19 infection. Thus, SARS-CoV-2 infection may be a cause of OME, and OME is a possible symptom of COVID19^7^.

OME has the characteristic of high prevalence in winter and spring, and is more common in children, mainly due to the anatomic and functional immaturity of the eustachian tube in children compared to adults^4,8^, but forced isolation of children at home during SARS-CoV-2 pandemic cut down the incidence of OME in children^4,8,9^.

As China has previously taken comprehensive closure measures, the lifting of COVID-19 restrictions started from December 7, 2022. The change led to a unique phenomenon in the world, that is, almost people has been COVID-19 positive during followed 2 months. The number of positive people reached its peak on December 22 (6.94 million/day) in China^10^, But, as the northeast region of China, all residential areas of Shenyang city were fully unsealed on December 14, 2022. Recently, two systematical reviews have underscored the need for standardized diagnostic protocols and large-scale multicenter research to clarify pathophysiology and optimized management of COVID-19-related otologic and neurologic manifestations^11,12^. They clearly indicate that olfactory dysfunction in COVID-19 often precedes or accompanies other symptoms, with objective testing revealing higher prevalence than self-report, especially for female predominance with an incidence of 34–68%, and sudden sensorineural hearing loss (SSNHL) in COVID-19 often accompanies tinnitus symptoms with an incidence of 61%, which is related to the impaired audio-vestibular system by SARS-CoV-2. Glucocorticoids remain the mainstay of treatment. Also, from December 2022 to March 2023, the OME incidence in our emergency and outpatient department increased significantly compared with the incidence in the same month before the epidemic. COVID-19 infection may increase severity of mental pressure among survivors^10,11^, ^13,14^. But, since Chinese people experienced the baptism of SARS in 2003, and Chinese government took full closure measures when facing COVID-19. Individuals with COVID-19 infection were isolated and actively treated^15^. The severity of the epidemic has brought little physiological and psychological pressure among Chinese people, but the severity of isolated closure may mainly induce mental stress from home and work.

However, the association of socioeconomic status (SES) and OME may be mediated by psychological distress moderated the mediation effect^16,17^. Thus, the aim of this study was to analyze whether COVID-19 infection may increase the risk of OME, especially in children, and whether low SES may increase the risk of OME caused by COVID-19 infection, especially in the first month following COVID-19 infection in Northeast China.

## Materials and methods

### OME patients

We conducted a retrospective case-crossover study in a single-center (First Affiliated Hospital of China Medical University) located in Shenyang (the capital city of Liaoning province). All protocols were approved by the medical ethics committee of First Affiliated Hospital of China Medical University. By the end of 2024, the permanent resident population of Shenyang reached 9.243 million, while the total number of outpatient and emergency visits at this center amounted to 3.618 million. Patients were diagnosed by otolaryngologists in the Department of Otolaryngology. According to the corresponding ICD-10 code (H65: OME), the specialized doctors needed to log into the outpatient and emergency treatment systems, and collect the patient information about OME symptoms. OME was defined as an independent clinical entity differentiated from acute otitis media and chronic suppurative otitis media. It is characterized by the presence of middle ear effusion without the acute inflammation typical of acute otitis media, nor the clinical manifestations of tympanic membrane perforation and intratympanic purulent discharge seen in chronic suppurative otitis media. For demographic indicators, we collected them through a telephone one-to-one questionnaire after the diagnosis and troubleshooting. The OME symptoms included one or both ear tightness, hearing loss, etc. Physical examination included one or both sides of the eardrum had invagination, fluid lines, and an amber color. Pure tone audiometry showed unilateral or bilateral conductive hearing loss, and tympanometry (acoustic impedance examination) showed a "B" or "C"—shaped curve. We recorded the number and symptoms (dry cough, dyspnea, sore throat, smell/taste disturbances, vertigo, dizziness, nausea and vomiting, sudden unilateral loss of hearing, progressive loss of hearing, and tinnitus) of OME patients by diagnosed in the ear nose throat clinics, from the same time periods pre- (15 December 2018–15 February 2019), during (15 December 2022–15 February 2023), and post- (15 December 2023–15 February 2024) the epidemic.

### Exposure assessment

In the early stage of COVID-19 outbreak (from December 2019 to January 2020), COVID-19 mainly affected cities in Hubei Province, but China quickly took measures to close its homes, so there was no outbreak of COVID-19 in cities of Liaoning Province. Starting from December 14, 2022, when the lockdown was lifted, there was a trend of outbreaks and even epidemics in Liaoning Province. Specially, all of patients who came to the emergency and outpatient during the Chinese epidemic period (15 December 2022–15 February 2023) had negative test certificate of COVID-19 at enrollment. These patients either previously positive for SARS-CoV-2 in a polymerase chain reaction (PCR) test or an antigen test from a nasopharyngeal swab 2–4 weeks ago, or had never suffered from it. COVID-19 exposure was assigned based on OME’s SARS-CoV-2 antigen detection for each of the same day (lag0) and one week before (lag1). Exposure was parameterized as either a status with (i) COVID-19 infection, or (ii) no COVID-19 infection. For each OME patient, the diagnosis date was used as the case day. The control days were defined as days occurring in the same month. The COVID-19 symptoms included fever, sore throat, dry cough, smell/taste disturbances, farting/diarrhea, fatigue, asthma, rash, nausea and vomiting, loss of appetite, tinnitus, dizziness, ear pain and tightness, sudden unilateral loss of hearing, progressive loss of hearing, and tinnitus.

### SES assessment

Based on our previous studies^18,19^, we established a comprehensive scoring system of SES, which was composed of the education, occupation and annual household income. This study used a comprehensive scoring system of SES, which is composed of the occupation and education level of included patients, and income in the past year. Occupation was divided into three grades and scores, including manual workers, farmers, unemployed people (low, 1 point), businessmen or employees (middle, 2 points), professionals, managers or government employees (high, 3 points). Education was divided into three grades and scores, including lower than high school (low, 1 point), high school graduation or equivalent (middle, 2 points), university graduation or above (high, 3 points). Annual household income (unit: RMB) also was divided into three grades and scores, including low (< 5000/month, 1 point), middle (5000–8000/month, 2 points), and high (8000–/month, 3 points). Then, the total SES scores were from 3 to 9 points by adding three scores. The terciles of SES status were divided into 3 levels including low (≤ 4 points), middle (5–7 points), and high (≥ 8 points).

### Statistical analysis

We used EpiData 4.0 software to enter data in the form of double entry, and finally selected 10% of the questionnaires for review. SPSS 20.0 and R 4.2.2 software were used for statistical analysis on the data. Firstly, we used a quasi-experimental analysis (an interrupted time series regression) within the framework of Poisson generalized additive model to assess the impact of COVID-19 outbreak on the emergency and outpatient for OME. This intermittent time series regression was: *Log* (*Y*_*t*_) = *β*_0_ + *β*_1_ × *time*_*t*_ + *β*_2_ × *outbreak*_*t*_ + *β*_3_ × *time after outbreak*_*t*_ + *s* (*status*, *df*) + *ε*_*t.*_ Among them, *Y*_*t*_ was the monthly emergency and outpatient count for OME. *β*_0_ was the baseline mean level of outpatient OME at time 0. *β*_1_ was the change in emergency and outpatient for OME (*Y*_*t*_) associated with an increase per unit time. *Outbreak*_*t*_ was the Chinese epidemic period (15 December 2022–15 February 2023, as mentioned above), which was a binary variable of “0” (pre-epidemic), “1” (during epidemic), and “2” (post- epidemic). *β*_2_ was the immediate change of average counts of emergency and outpatient for OME during and post-epidemic (immediate change). *β*_3_ was the trend change during and post-epidemic. *S* (status, *df*) was the cubic spline of status variables (genetics, allergic constitution, diet, SES) with corresponding degrees of freedom (df) after adjusting confounding effects. *ε*_t_ was the residual series. We used the lowest value of the generalized cross-validation results as the optimal model (Supplemental Table 1).

Secondly, we used a time stratified case crossover study design with a conditional logistic regression to examine the associations between COVID-19 infection and the incidence of OME in Shenyang from 2018 to 2024 (December of that year to February of the following year), and the risk of emergency and outpatient for OME associated with SES would be further induced after COVID-19 infection. The date of emergency and outpatient for OME was defined as the case day. The same day in other years was chosen as the control day. The modification effects were evaluated by subgroup, including symptoms of COVID-19 (respiratory and digestive), gender (male and female), and age (≤ 50 years and > 50 years). We used a lag up (one year) to fit the lag-response associations between SES and emergency and outpatient for OME, with a smoothing function of natural cubic splines to adjust for non-linear effects (symptoms, gender and age) on OME, and used high SES as the reference status to quantify the effect of SES on OME (due to the minimum odds ratio) (Supplemental Table 2). The lowest Akaike Information Criterion (AIC) value expressed the optimal model (Supplemental Table 3).

Thirdly, because Chinese government lifted home lockdown from December 14, 2022, the COVID-19 had broken out in Liaoning Province. We further evaluated the disparity of SES effect in the first three months following the Chinese special outbreak period (15 December 2022–15 February 2023). The COVID-19 epidemic severely hindered the development of the global economy, the SES levels of most households declined from January 2020 to March 2024 in Shenyang (Table 1). We estimated the risk of emergency and outpatient for OME associated with SES for the first three months during the epidemic outbreak.

**Table 1:** Characteristics of emergency and outpatient cases with OME and status variables in Liaoning during three periods.

Variables	Three period	Pre-epidemic	during-epidemic	post- epidemic
All	1080	187	568	325
Gender* n* (%)				
Male	572 (53.00)	95 (50.80)	302 (53.17)	175 (53.85)
Female	508 (47.00)	92 (49.20)	266 (46.83)	150 (46.15)
Age* n* (%)				
≤ 50 years	548 (50.74)	95 (50.80)	287 (50.53)	166 (51.08)
> 50 years	532 (49.26)	92 (49.20)	281 (49.47)	159 (48.92)
Accompanying symptoms* n* (%)				
Respiratory symptoms	584 (54.07)	104 (55.61)	309 (54.40)	171 (52.62)
Digestive symptoms	204 (18.89)	33 (17.65)	107 (18.84)	64 (19.69)
No	244 (27.04)	50 (26.74)	152 (26.76)	90 (27.69)
Status variables *n* (%)				
Low SES	620 (57.41)	90 (48.13)	346 (60.92)	184 (56.62)
Vegetarian diet	148 (13.70)	26 (13.90)	77 (13.56)	45 (13.85)
Allergic constitution	292 (27.04)	49 (26.20)	157 (27.64)	86 (26.46)
Genetics	176 (16.30)	32 (17.11)	92 (16.20)	52 (16.00)

pre- (15 December 2018–15 February 2019), during (15 December 2022–15 February 2023), and post- (15 December 2023–15 February 2024) the epidemic.

The accompanying symptoms included respiratory symptoms (fever, sore throat, dry cough, nausea) and digestive symptoms (smell/taste disturbances, farting/diarrhea, nausea and vomiting, loss of appetite).

Then, the model robustness was assessed by sensitivity analysis. We found changes in the smoothness of the cubic spline of model status variables (Supplemental Table 1, 4). Diets may be a confounder between SES and OME based on previous studies^17–22^. But, according to the AIC value, the diets model was not the best model in this study. So, we added “allergic constitution” and “genetics” to the main model of SES, and then compared the exposure response curve relationship for sensitivity analysis (Supplemental Table 5).

## Results

### Population characteristics

We collected 1080 (male 53%; female 47%) cases of emergency and outpatient for OME from three periods. The mean [SD] age was 47.57 [15.27] in male, and 47.80 [17.66] in female. Among symptoms accompanying otitis media, respiratory symptoms were mostly reported (54.07%), followed by digestive system symptoms (18.89%), no these symptoms (27.04%). The proportion of low SES and vegetarian diet, allergic constitution, genetics were 57.41%, and 13.70%, 27.04%, 16.30%, respectively (Table 1). Notably, the proportion of low SES increased by 12.79%, 8.49% during (15 December 2022–15 February 2023), after (15 December 2023–15 February 2024) than pre-the epidemic (15 December 2018–15 February 2019). The other state variables (vegetarian diet, allergic constitution, and genetics) remained unchanged overall.

### The COVID-19 outbreak increased emergency and outpatient for OME

When the lockdown was lifted, there was a trend of COVID-19 outbreaks and even epidemics in Liaoning Province. The risk of emergency and outpatient for OME suddenly increased during the epidemic (15 December 2022–15 February 2023). The estimates of the regression model showed a 50% higher risk (RR: 1.563, 95% CI 1.256–1.942) in emergency and outpatient for OME during the epidemic, corresponding to an increase of 127 OME patients per month compared with that pre-the epidemic, 81 OME patients per month compared with that post-the epidemic (Table 1 and 2).

**Table 2:** The estimates for interrupt time series regression.

	Variable	Beta	SE	RR (95% CI)	*P*-value
Emergency and outpatient	Epidemic (level)	0.446	0.111	1.563 (1.256, 1.942)	< 0.001
	Time pre-epidemic (trend)	0.024	0.049	1.025 (1.013, 1.036)	0.026
	Time post-epidemic (trend)	0.030	0.084	1.033 (1.024, 1.041)	0.032

### Short-term association between SES and the risk of OME

Table 3 showed the exposure–response relationships between SES and the risk of emergency and outpatient for OME at lag 0 (i.e., the same day of exposure) pre-, during, and post-the epidemic.

**Table 3:** Associations between SES and risks of emergency and outpatient for OME pre-, during, and post-epidemic.

		Pre-	During	Post-
Total cases	Low SES	1.266 (1.038, 1.596)	1.864 (1.295, 2.254)	1.196 (0.939, 1.514)
OR (95% CI)	Moderate SES	1.101 (0.897, 1.350)	1.675 (1.211, 2.127)	1.081 (0.882, 1.324)
	High SES	1.000 (0.998, 1.002)	1.000 (0.997, 1.003)	1.000 (0.998, 1.002)
Respiratory symptoms	Low SES	1.197 (0.978, 1.565)	1.205 (1.017, 1.427)	1.210 (1.020, 1.433)
OR (95% CI)	Moderate SES	1.063 (0.895, 1.264)	1.106 (0.893, 1.364)	1.064 (0.895, 1.265)
	High SES	1.002 (0.999, 1.005)	1.000 (0.998, 1.002)	0.999 (0.997, 1.001)
Digestive symptoms	Low SES	1.086 (0.869, 1.357)	1.163 (0.794, 1516)	1.031 (0.874, 1.215)
OR (95% CI)	Moderate SES	0.943 (0.765, 1.163)	1.064 (0.895, 1.265)	0.968 (0.777, 1.207)
	High SES	0.999 (0.997, 1.001)	1.000 (0.998, 1.002)	1.000 (0.997, 1.003)
Male	Low SES	1.119 (0.919, 1.323)	1.167 (0.892, 1.283)	1.021 (0.822, 1.269)
OR (95% CI)	Moderate SES	1.036 (0.880, 1.220)	1.050 (0.954, 1.387)	0.919 (0.723, 1.168)
	High SES	0.999 (0.997, 1.001)	1.000 (0.999, 1.001)	0.999 (0.997, 1.001)
Female	Low SES	1.125 (0.635, 2.146)	1.160 (0.865 1.719)	1.063 (0.481, 2.328)
OR (95% CI)	Moderate SES	1.023 (0.346, 1.952)	0.992 (0.206, 2.037)	1.141 (0.598, 2.136)
	High SES	1.000 (0.998, 1.002)	1.000 (0.998, 1.002)	1.000 (0.997, 1.003)
≤ 50 years	Low SES	1.164 (0.886, 1.594)	1.764 (1.390, 2.315)	1.184 (1.002, 2.217)
OR (95% CI)	Moderate SES	1.138 (0.632, 1.715)	1.235 (1.087, 2.265)	1.205 (1.017, 1.542)
	High SES	1.000 (0.998, 1.002)	1.000 (0.998, 1.002)	0.999 (0.997, 1.001)
> 50 years	Low SES	1.094 (0.911, 1.915)	1.178 (0.220, 1.514)	1.093 (0.728, 2.190)
OR (95% CI)	Moderate SES	1.081 (0.952, 1.609)	1.195 (0.539, 2.116	1.181 (0.930, 1.457)
	High SES	1.000 (0.998, 1.002)	1.000 (0.999, 1.001)	1.000 (0.998, 1.002)

For all OME cases, either pre- or post-the epidemic, low SES was associated with an increased risk of emergency and outpatient for OME, and during the epidemic, a higher risk appeared than pre- or post-the epidemic. By cause-specific analysis, no association for accompanying symptoms was found among three-stage (pre-, during, post-epidemic). For gender and age subgroups, the risk of emergency and outpatient for OME associated with low SES generally increased among three-stage (pre-, during, post-epidemic) and the most risk appeared during the epidemic. A higher risk was observed for OME patients (≤ 50 years) during the epidemic.

### Sensitive analysis

The similar results were shown in altering smoothing degrees in natural cubic splines of covariates by sensitivity analysis (Supplemental Table 1, 4, 5). During three periods (pre-, during, and post- epidemic), the patterns remained in SES-related emergency and outpatient for OME after adjusting for diets, which indicated our modeling robustness.

## Discussion

After COVID-19 infection, OME issue has not received widespread attention. Although SARS-CoV-2 exist in middle ear fluid^7,23,24^, the causal relationship cannot be verified. Previous studies also indicated that the incidence of OME in children decreased during the outbreak of COVID-19^4,8,9^, but this was contrary to our results, which can be related to the discrepancy of different time periods of the surveys and target populations. A retrospective descriptive study in China indicated the incidence of adult OME decreased during the closed period of home confinement, increased during the outbreak period, and recovered during the later stages of the pandemic, which suggested a link between COVID-19 and OME onset in the short term^24^. This study further revealed that a surge in the incidence of OME increased nearly 1–2 months post-Omicron infection by a quasi-experimental analysis, and a slow upward trend emerged one year after the outbreak of the epidemic. This study might highlight the potential link between SARS-CoV-2 and OME onset.

We found for the first time that low SES was associated with an increased risk of OME across the population before the COVID-19 pandemic (15 December 2018–15 February 2019) in Liaoning province as a representative of Northeast China, which has a population of 8.32 million and an area of 12,948 km^2^. This trend may be explained by three reasons. Firstly, low, moderate and high SES seemed to have a graded effect on OME. The COVID-19 epidemic had a huge impact on the global economy, and in China, the city lockdown policy or home confinement led to an increasing of low SES. Secondly, these low SES families may have the poor hygiene standards and poor nutritional conditions^16^. Thirdly, low SES families may also increase the mental stress, which became even more severe after the outbreak of the epidemic^25^. And this mental stress status may weaken the body’s immunity. The low-income individuals could not get health-insurance during the epidemic period, which made it impossible to guarantee their personal health status, while reducing the labor supply rate, especially for young men^26^

For subgroup analysis, individuals under 50 years old had a higher risk of OME related to SES during the epidemic than that pre- and post-epidemic in Liaoning. However, a descriptive study conducted during the same period in Anhui found that the elderly were more prone to OME during the epidemic. This inconsistency may be related to the difference in investigation methods and locations. That previous study collected data from Anhui province, which is adjacent to the origin of the epidemic in Hubei province. The elderly in Anhui had more persistent residual effects of the novel coronavirus on the eustachian tube^24^. However, Liaoning is far away from Hubei Province. After the discovery of COVID-19 in Hubei, China launched a series of preventive measures including school and business closures, and home confinement. As a result, the economic burden and sense of family may be more prone to worsen individuals under 50 years old than the elderly. Moreover, Liaoning is a heavy industrial area with a backward economy in China. More importantly, our results further showed low SES increased the incidence of OME at three months following the COVID-19 outbreak and the greatest risk occurred in January 2023 (the first month after COVID-19 infection in China). Notably, SES suddenly decreased from the non-pandemic period to pandemic period, which indicated high SES might decrease the risk of OME after COVID-19 infection.

The included cases strictly conformed to the clinical diagnostic criteria for OME rather than acute otitis media or the recovery phase of acute otitis media. Given that both eustachian tube dysfunction and local middle-ear inflammation/infection are implicated in OME development, SES and COVID-19 infection may participate in OME pathogenesis through multiple interconnected pathways. Lower socioeconomic status is often associated with inadequate access to healthcare, poor nasal and upper respiratory tract health, and higher exposure to respiratory pathogens, all of which can impair Eustachian tube function and trigger local inflammatory responses in the middle ear. Similarly, COVID-19 infection primarily targets the upper respiratory tract, potentially inducing mucosal inflammation, edema, and functional disturbance of the eustachian tube, as well as promoting local inflammatory or infectious processes in the middle ear cavity. These mechanisms collectively provide a reasonable biological basis for the observed associations in this study.

This study had several limitations. First, the included individuals were from a single center from Liaoning, as a heavy industrial and economically backward area in Northeast China. Second, we did not analyze the effect of specific components in SES on the risk of OME. Third, the quasi-experimental analysis did not control the confounding factor, that was, COVID-19-related accompanying symptoms which might affect OME after COVID-19 infection. Fourth, we did not control COVID-19 confounding effect on SES related OME with the insufficient medical service data after COVID-19 infection. Supplemental Table 6 showed the comparison of other related studies with this study during the pandemic.

## Conclusion

As unexpected, after COVID-19 infection, the risk of emergency and outpatient for OME suddenly increased to three times of the same period in our center, a single hospital in Shenyang from Northeast China. To our knowledge, this study was the first to prove that low SES strengthened the adverse effect of emergency and outpatient for OME, and further provided evidence that low SES may increase the risk of emergency and outpatient for OME after COVID-19 infection, with the greatest risk increase occurring 1–2 months (15 December 2022–15 February 2023) after COVID-19 infection. Specially, people aged less than 50 years old were vulnerable to low SES.

## Supplementary Information

Below is the link to the electronic supplementary material.

Supplementary Material 1.

## Data Availability

The datasets used and/or analyzed during the current study were available from the corresponding author on reasonable request.
