# Supplementary material for: Lower socioeconomic status strengthens the risk of otitis media with effusion after COVID-19 infection: a case-crossover study in Northeast China
Source: Sci Rep. 2026 Jul 9;16:24171. doi: 10.1038/s41598-026-61832-9 (PMC13442866; doi:10.1038/s41598-026-61832-9)
Supplement: Supplementary file 1 [file 41598_2026_61832_MOESM1_ESM.docx]

**Supplemental Table 1**

The optimal model of interrupt time series regression for otitis media with effusion by generalized additive model.

| Outpatient department visits for OME | *df* =3, GCV | *df* =4, GCV | *df* =5, GCV | *df* =6, GCV |
| --- | --- | --- | --- | --- |
| time + outbreak + time after outbreak + SES | 2.14 | 2.14 | 2.15 | 2.15 |
| time + outbreak + time after outbreak + SES + diet | 2.16 | 2.17 | 2.18 | 2.18 |
| time + outbreak + time after outbreak + SES + diet + allergic constitution | 2.19 | 2.20 | 2.15 | 2.15 |
| time + outbreak + time after outbreak + SES + diet + allergic constitution + genetics ^*^ | 2.23 | 2.24 | 2.10 | 2.11 |

OME otitis media with effusion, SES socioeconomic status, *df* degree of freedom, GCV generalized cross-validation.

The optimal model was determined by the lowest value of GCV.

^*^ the optimal model.

**Supplemental Table 2**

Associations between SES and risks of emergency and outpatient for OME at lag 0 pre-, during, and post-epidemic by accompanying symptoms^*^ of COVID-19, gender, and age.

|  |  | Pre- | During | Post- |
| --- | --- | --- | --- | --- |
| Total cases | Low SES | 1.210 (0.803, 1.726) | 1.815 (1.157, 2.925) | 1.169 (0.626, 2.192) |
| OR (95% CI) | Moderate SES | 1.135 (0.852, 1.369) | 1.640 (1.070, 2.230) | 1.214 (0.754, 2.951) |
|  | High SES | 0.999(0.998, 1.000) | 1.001 (0.999, 1.002) | 0.999(0.997, 1.001) |
| Respiratory symptoms | Low SES | 1.326(0.334, 3.985) | 2.113(1.299, 7.143) | 1.256(0.863, 2.527) |
| OR (95% CI) | Moderate SES | 1.027 (0.136, 3.158) | 1.874 (1.115, 3.127) | 1.263 (0.205, 2.751) |
|  | High SES | 1.000(0.996, 1.003) | 0.999(0.997, 1.001) | 0.999 (0.996, 1.001) |
| Digestive symptoms | Low SES | 0.984 (0.463, 1.210) | 1.224 (0.618, 2.057) | 1.216 (0.758, 1.512) |
| OR (95% CI) | Moderate SES | 0.818 (0.672, 0.956) | 1.164 (0.883, 1.400) | 1.208 (0.770, 1.980) |
|  | High SES | 1.001 (1.000, 1.002) | 1.000 (0.999, 1.001) | 1.000(0.999, 1.000) |
| Male | Low SES | 1.019 (0.663, 1.376) | 1.302 (0.793, 2.101) | 0.894 (0.385, 1.728) |
| OR (95% CI) | Moderate SES | 0.945 (0.410, 1.502) | 1.211(0.405, 3.303) | 1.039 (0.579, 2.026) |
|  | High SES | 0.999(0.997, 1.001) | 1.000 (0.999, 1.000) | 0.999 (0.998, 1.000) |
| Female | Low SES | 1.125 (0.635, 2.146) | 1.188 (0.868 1.729) | 0.952 (0.354, 2.240) |
| OR (95% CI) | Moderate SES | 1.009 (0.253, 1.905) | 0.992 (0.206, 2.037) | 1.208(0.687, 2.124) |
|  | High SES | 1.000(0.999, 1.000) | 0.999 (0.997, 1.001) | 0.999 (0.998, 1.001) |
| ≤50 years | Low SES | 1.023 (0.601, 2.109) | 1.301 (0.983, 2.356) | 1.071 (0.252, 2.049) |
| OR (95% CI) | Moderate SES | 1.011 (0.516, 2.165) | 1.275 (0.841, 2.124) | 0.979 (0.313, 1.872) |
|  | High SES | 1.001 (0.999, 1.002) | 1.000(0.999, 1.001) | 0.999(0.998, 1.000) |
| >50 years | Low SES | 1.151 (0.275, 1.803) | 1.021 (0.305, 2.049) | 1.128 (0.279, 1.998) |
| OR (95% CI) | Moderate SES | 1.010 (0.242, 2.163) | 1.176 (0.523, 2.029) | 0.989 (0.275, 1.803) |
|  | High SES | 0.999 (0.998, 1.000) | 1.000(0.999, 1.001) | 0.999 (0.998, 1.001) |

*The accompanying symptoms included respiratory symptoms (fever, sore throat, dry cough, nausea) and digestive symptoms (smell/taste disturbances, farting/diarrhea, nausea and vomiting, loss of appetite).

**Supplemental Table 3**

Associations between SES and risks of emergency and outpatient for OME at lag 1 pre-, during, and post-epidemic by accompanying symptoms^*^ of COVID-19, gender, and age.

|  |  | Pre- | During | Post- |
| --- | --- | --- | --- | --- |
| Total cases | Low SES | 1.115 (0.728, 1.516) | 1.793 (1.243, 2.211) | 1.202 (0.705, 2.024) |
| OR (95% CI) | Moderate SES | 1.044 (0.504, 1.242) | 1.581 (1.116, 2.153) | 1.183 (0.612, 2.951) |
|  | High SES | 1.000 (0.997, 1.002) | 0.999 (0.996, 1.003) | 1.000 (0.998, 1.002) |
| Respiratory symptoms | Low SES | 1.120 (0.315, 1.715) | 1.214 (1.083, 2.923) | 1.165 (0.776, 1.896) |
| OR (95% CI) | Moderate SES | 1.019 (0.128, 2.219) | 1.106 (0.503, 2.152) | 1.172 (0.294, 2.065) |
|  | High SES | 0.998 (0.995, 1.001) | 1.000 (0.996, 1.004) | 0.999 (0.997, 1.002) |
| Digestive symptoms | Low SES | 1.039 (0.684, 1.520) | 1.250 (0.511, 2.899) | 0.882 (0.215, 1.337) |
| OR (95% CI) | Moderate SES | 0.937 (0.763, 1.047) | 1.171 (0.675, 2.036) | 1.008 (0.885, 1.325) |
|  | High SES | 0.998 (0.995, 1.001) | 1.001 (0.998, 1.009) | 1.002 (0.997, 1.004) |
| Male | Low SES | 1.001 (0.872, 1.268) | 1.302 (0.793, 2.101) | 0.998 (0.552, 1.653) |
| OR (95% CI) | Moderate SES | 0.936 (0.371, 1.253) | 1.164 (0.376, 4.026) | 1.026 (0.436, 1.817) |
|  | High SES | 0.999 (0.998, 1.001) | 1.000 (0.999, 1.000) | 0.999 (0.998, 1.000) |
| Female | Low SES | 1.125 (0.635, 2.146) | 1.160 (0.865 1.719) | 1.063 (0.481, 2.328) |
| OR (95% CI) | Moderate SES | 1.023 (0.346, 1.952) | 0.992 (0.206, 2.037) | 1.141 (0.598, 2.136) |
|  | High SES | 1.001 (0.998, 1.004) | 0.999 (0.997, 1.001) | 1.000 (0.998, 1.003) |
| ≤50 years | Low SES | 1.212 (0.504, 3.128) | 1.306 (0.494, 2.643) | 1.205 (0.620, 3.224) |
| OR (95% CI) | Moderate SES | 1.198 (0.210, 1. 516) | 1.275 (0.841, 2.124) | 1.096 (0.334, 1.853) |
|  | High SES | 1.001 (0.999, 1.002) | 1.000 (0.998, 1.002) | 0.999 (0.997, 1.001) |
| >50 years | Low SES | 1.138 (0.277, 1.915) | 1.022 (0.220, 2.017) | 1.093 (0.222, 2.190) |
| OR (95% CI) | Moderate SES | 1.142 (0.353, 1.895) | 1.034 (0.323, 3.318) | 1.043 (0.357, 2.017) |
|  | High SES | 0.999 (0.997, 1.001) | 1.000 (0.999, 1.001) | 0.999 (0.997, 1.001) |

^*^The accompanying symptoms included respiratory symptoms (fever, sore throat, dry cough, nausea) and digestive symptoms (smell/taste disturbances, farting/diarrhea, nausea and vomiting, loss of appetite).

**Supplemental Table 4**

The optimal model of case cross-over analysis for OME.

| Model |  |  | *df* and AIC |  |
| --- | --- | --- | --- | --- |
| Outpatient department visits for OME | *df*=3, AIC | *df*=4, AIC | *df*=5, AIC | *df*=6, AIC |
| SES + allergic constitution ^*^ | 2850.792 | 2859.481 | 2871.536 | 2878.843 |
| SES + genetics | 2860.110 | 2868.853 | 2878.218 | 2881.410 |
| SES + allergic constitution + genetics | 2869.793 | 2871.083 | 2881.424 | 2888.530 |
| SES + diet | 2870.343 | 2868.508 | 2869.767 | 2875.032 |

AIC Akaike Information Criterion, *df* degree of freedom, OME otitis media with effusion, SES socioeconomic status.

The optimal model was determined by the lowest value of AIC.

^*^ the optimal model.

**Supplemental Table 5**

Associations between SES and risks of emergency and outpatient for OME pre-, during, and post-epidemic in subgroups, adjusting diets.

|  |  | Pre- | During | Post- |
| --- | --- | --- | --- | --- |
| Total cases | Low SES | 1.072 (0.326, 1.564) | 1.136 (0.474, 1.359) | 1.148 (0.533, 2.473) |
| OR (95% CI) | Moderate SES | 1.038 (0.549, 1.259) | 1.074 (0.761, 1.515) | 1.064 (0.752, 1.505) |
|  | High SES | 0.998 (0.996, 1.000) | 0.999 (0.997, 1.001) | 1.000 (0.997, 1.003) |
| Respiratory symptoms | Low SES | 1.010 (0. 991, 1.029) | 1.083 (0.517, 1.316) | 1.169 (0.437, 1.716) |
| OR (95% CI) | Moderate SES | 1.023 (1.000, 1.047) | 1.026 (0.589, 1.787) | 1.119 (0.783, 1.601) |
|  | High SES | 0.999 (0.997, 1.001) | 0.999 (0.998, 1.000) | 0.998 (0.997, 0.999) |
| Digestive symptoms | Low SES | 1.016 (0.711, 1.453) | 1.308 (0.797, 1.867) | 1.015 (0.853, 1.483) |
| OR (95% CI) | Moderate SES | 0.977 (0.958, 0.996) | 1.087 (0.870, 1.625) | 1.088 (0.590, 1.784) |
|  | High SES | 1.000 (0.997, 1.003) | 1.001 (0.999, 1.003) | 0.999 (0.997, 1.002) |
| Male | Low SES | 1.203 (0.829, 1.744) | 1.299 (0.456, 2.985) | 0.991 (0.629, 1.560) |
| OR (95% CI) | Moderate SES | 0.972 (0.669, 1.338) | 1.144 (0.919, 1.770) | 1.080 (0.470, 1.364) |
|  | High SES | 1.000 (0.998, 1.002) | 1.000 (0.999, 1.001) | 1.000 (0.999, 1.001) |
| Female | Low SES | 1.175 (0.746, 1.850) | 1.666 (0.767, 3.621) | 1.230 (0.829, 1.823) |
| OR (95% CI) | Moderate SES | 1.057 (0.432, 1.588) | 0.982 (0.428, 2.254) | 1.059 (0.718, 1.560) |
|  | High SES | 1.008 (0.988, 1.028) | 0.997 (0.995, 0.999) | 1.003 (0.992, 1.010) |
| ≤50 years | Low SES | 1.069 (0.275, 1.736) | 1.381 (0.611, 3.193) | 1.314 (0.120, 3.810) |
| OR (95% CI) | Moderate SES | 0.993 (0.972, 1. 014) | 1.319 (0.547, 3.185) | 1.096 (0.304, 2.702) |
|  | High SES | 1.000 (0.998, 1.002) | 1.000 (0.999, 1.001) | 1.000 (0.998, 1.002) |
| >50 years | Low SES | 1.206 (0.802, 1.813) | 1.292 (0.740, 2.131) | 1.211 (0.860, 1.704) |
| OR (95% CI) | Moderate SES | 1.061 (0.752, 1.500) | 1.146 (0.722, 1.940) | 0.985 (0.426, 2.276) |
|  | High SES | 1.000 (0.998, 1.002) | 1.000 (0.998, 1.002) | 0.999 (0.996, 1.002) |

**、**

**Supplemental Table 6** Summary other related studies during the pandemic and comparison with this study

| Study | Country/Population | Key Findings | Comparison with this study |
| --- | --- | --- | --- |
| [4] Iannella et al. (2022)  Eur Arch Otorhinolaryngol | Italy/ adults and children | OME incidence decreased in children during lockdown but increased in adults post-lockdown. SARS-CoV-2 may be a trigger for OME in adults | We found an overall increase in OME during the outbreak period, including in adults, consistent with their adult findings. However, we did not observe a decrease in children possibly due to differences in lockdown timing and age distribution. |
| [9] Hullegie et al. (2021) Front Cell Infect Microbiol | Netherlands/children | Strong decline in childhood OME during COVID-19 restrictions, likely due to reduced viral transmission | We did not focus exclusively on children, but overall OME cases increased. We found that individuals under 50 years (including younger adults) were more vulnerable to low SES related OME risk, contrasting with the pediatric specific decline reported in this study. |
| [10] Yu et al. (2023)  Front Public Health | China/children | Social distancing during lockdown reduced acute otitis media in children. | This study covered a later period (2022–2024) when restrictions were lifted, leading to a surge in infections, and reflected the post-lockdown rebound, not the lockdown period itself. |
| [25] Qi et al. (2024)  Eur Arch Otorhinolaryngol | China/adults | OME incidence in adults decreased during lockdown, increased during outbreak, and recovered later. Older adults were more affected | We similarly observed a surge post-lockdown, but found that individuals ≤50 years had the highest SES-related risk. This age discrepancy may reflect regional differences (Liaoning vs. Anhui) in population structure, economic stress, or viral strain timing. |
| [7] Fan et al. (2023)  Int J Infect Dis | China/COVID-19 patients | SARS-CoV-2 detected in middle ear fluid of OME patients, which showed direct viral involvement. | We did not test MEF for SARS-CoV-2, but provided epidemiological evidence supporting a temporal association between COVID-19 infection and OME onset, especially in low SES groups. |
| [24] Han et al. (2024)  Am J Otolaryngol | China/OME patients post-Omicron | SARS-CoV-2 detected in MEF; OME may be a sequela of Omicron infection. | We found increased OME risk in the 1–2 months following Omicron-dominant outbreak, reinforcing the post-infection temporal link. |

OME otitis media with effusion, SES socioeconomic status
